# Paroxysmal Laryngospasm: A Rare Condition That Respiratory Physicians Must Distinguish from Other Diseases with a Chief Complaint of Dyspnea

**DOI:** 10.1155/2020/2451703

**Published:** 2020-07-04

**Authors:** Yu Bai, Xi-Rui Jing, Yun Xia, Xiao-Nan Tao

**Affiliations:** ^1^Department of Respiratory and Critical Care Medicine, Union Hospital, Tongji Medical College, Huazhong University of Science and Technology, Wuhan, Hubei 430022, China; ^2^Department of Orthopedics, Union Hospital, Tongji Medical College, Huazhong University of Science and Technology, Wuhan, Hubei 430022, China; ^3^Department of Nephrology, The First People's Hospital of Jiangxia District, Wuhan, Hubei 430022, China

## Abstract

**Background:**

In recent years, we have observed respiratory difficulty manifested as paroxysmal laryngospasm in a few outpatients, most of whom were first encountered in a respiratory clinic. We therefore explored how to identify and address paroxysmal laryngospasm from the perspective of respiratory physicians.

**Methods:**

The symptoms, characteristics, auxiliary examination results, treatment, and prognosis of 12 patients with paroxysmal laryngospasm treated in our hospital from June 2017 to October 2019 were analyzed.

**Results:**

Five males (42%) and 7 females (58%) were among the 12 Han patients sampled. The average age of the patients was 49.25 ± 13.02 years. The disease course ranged from 14 days to 8 years and was characterized by sudden dyspnea, an inability to inhale and exhale, a sense of asphyxia, and voice loss during an attack. Eight patients with gastroesophageal reflux were cured after antacid treatment. One case of upper respiratory tract infection (URI) was completely relieved after symptomatic treatment. One patient with left vocal cord paralysis experienced complete relief after specialist treatment by an otorhinolaryngologist. Episodes in 1 patient were significantly reduced after lifestyle improvement. One patient experienced spontaneous relief after rejecting treatment.

**Conclusions:**

Paroxysmal laryngospasm is a rare laryngeal disease that generally occurs secondary to gastroesophageal reflux disease (GERD), and antireflux therapy is frequently effective for its treatment. A respiratory physician should master and identify the symptoms and differentiate this condition from hysterical stridor, reflux-related laryngospasm, and asthma. Timely referral to otolaryngologists, gastroenterologists, and other specialists for standardized examination and regular treatment should be provided when necessary.

## 1. Introduction

Dyspnea is a common clinical symptom with several well-defined causes: pulmonary dyspnea, cardiogenic dyspnea, dyspnea caused by hematologic abnormalities, central nervous system dyspnea, dyspnea caused by endocrine abnormalities, and dyspnea associated with hysteria [1, 2]. Dyspnea caused by various conditions has its own distinct characteristics [3]. However, in recent years, we have observed respiratory difficulty manifested by paroxysmal laryngospasm in a few outpatients. Most of these patients have severe dyspnea during an attack. Several patients cannot obtain a definite diagnosis and treatment. In contrast to respiratory physicians, otolaryngologists and anesthesiologists are experts in managing paroxysmal laryngospasm. Articles related to this condition are also published in otolaryngology, anesthesiology, and other specialized journals. We therefore urge pulmonologists to understand and become familiar with paroxysmal laryngospasm in order to improve the management of this condition.

Laryngospasm, a clinical symptom characterized by involuntary laryngeal muscle spasm, is a manifestation of glottic obstruction when vocal cords are closed. Vocal cords and soft tissue of the supraglottic folds are blocked at the upper airway, resulting in obstruction of inspiration and expiration, which sometimes occurs during or after the administration of anesthesia and is associated with severe perioperative complications. Failure to manage this condition leads to hypoxia, hypercapnia, bronchospasm, pulmonary edema, arrhythmia, and heart failure, among other sequelae, which can eventually cause death from severe laryngeal spasm [4, 5]. One type of reactive airway obstruction is paroxysmal laryngospasm, which is a rare laryngeal disease in adults. In this condition, the throat is completely closed due to some form of hypersensitivity or a protective laryngeal reflex causing a transient, complete inability to breathe. Paroxysmal laryngospasm onset in patients is often characterized by a sudden and complete inability to breathe, along with voice loss or hoarseness and stridor. Paroxysmal laryngospasm usually lasts from several seconds to several minutes [6] and may be accompanied by obvious causes such as upper respiratory tract infection (URI), emotional agitation or tension, and/or severe coughing.

Several studies have established that paroxysmal laryngospasm is often secondary to laryngopharyngeal reflux, a variant of gastroesophageal reflux disease (GERD). Paroxysmal laryngospasm is often misdiagnosed as asthma, hysterical stridor, obstructive sleep apnea, paroxysmal nocturnal dyspnea, and other conditions [7]. Patients with paroxysmal laryngospasm have a short attack period and often show no symptoms and signs after these episodes. The diagnosis usually relies on clinical manifestations [8]. Therefore, clinicians who do not understand the clinical manifestations of this condition regularly misdiagnose the disease [9]. Paroxysmal laryngospasm yields obvious dyspnea; consequently, this symptom should be recognized not only by otolaryngologists, anesthesiologists, and gastroenterology physicians but also by respiratory physicians. Therefore, we call on pulmonologists to be conversant with the management and treatment of paroxysmal laryngospasm and to refer patients to relevant specialists when necessary. Based on experience in our clinic, 12 cases of paroxysmal laryngospasm were reviewed and described in this work.

## 2. Methods

### 2.1. Participants

We collected data from 12 patients seen at the Department of Respiratory and Critical Care Medicine, Union Hospital Affiliated with Tongji Medical College, Huazhong University of Science and Technology, China, from June 2017 to October 2019. Patients were all from the Han tribe. Five of the patients were male, representing 42%, and 7 were female, representing 58%. The average age was 49.25 ± 13.02 years. There were 3 smokers and 2 drinkers. The course of the disease ranged from 14 days to 8 years. Neurological diseases and surgical history were excluded in this group. Five patients had severe coughing before their laryngospasm. Three patients had an antecedent URI, 2 had some form of emotional agitation before their laryngospasm, while 2 had no obvious provoking factors. Eleven patients stated that their symptoms occurred during the day, and only 1 had nocturnal symptoms.

### 2.2. Clinical Manifestations and Related Findings

Table 1 lists the concomitant conditions of all the patients. These patients came to see a doctor because they were experiencing sudden dyspnea, an inability to inhale and exhale, a sense of asphyxia, and loss of their voice during attacks. Two patients mostly complained of wheezing. The duration of each attack varied among the patients from a minimum duration of 10 seconds to a maximum of 2 minutes. All patients were able to recover from the attacks on their own. Eight of the 12 patients had a history of GERD. Seven of these had conscious sensation of regurgitation into the pharynx as part of their GI problems. All 12 patients had signs of chronic pharyngitis such as mucosal hyperemia and hypertrophic lymphoid follicles. Apart from these findings and history, these patients had no other relevant complaints or conditions. Their workup included pulmonary CT, pulmonary function testing, routine blood examination, and electrocardiograms, all of which were negative. All other fiberoptic laryngoscopy studies were within normal limits except for one case of left vocal cord paralysis identified.

## 3. Results

### 3.1. Diagnosis

Laryngeal function, particularly with respect to vocal fold movement, would be expected to be normal in patients with reflux-related paroxysmal laryngospasm as observed in this study with the exception of one patient with unilateral paralysis. Even the patients without GERD histories had normal fiberoptic exams. No patients underwent a fiberoptic exam during one of their laryngospasm events. The diagnosis of paroxysmal laryngospasm mainly depends on a patient's medical history and a lack of alternative explanations for symptoms. An alternative explanation was identified in only 1 of the 12 patients, and we diagnosed paroxysmal laryngospasm in the remaining 11 patients based on the following medical histories and clinical manifestations: (1) with or without obvious triggers, URI, emotional agitation or tension, and a history of GERD; (2) typical laryngospasm attacks: sudden dyspnea, asphyxiation, stridor, and hoarseness usually lasting for several seconds to several minutes; and (3) exclusion of asthma, hysterical stridor, obstructive sleep apnea-hypopnea syndrome, heart attack, epilepsy, rabies, tumor, nervous system disorders, and other diseases. Hysterical stridor was excluded because it has a strong demographic pattern of occurring in young adult females, lasting for minutes to hours, frequently requiring sedation or anxiolytics for treatment, and persisting for years. The patient with vocal fold paralysis in this series responded to surgical treatment by an ear-nose-throat (ENT) specialist.

### 3.2. Treatment

All eight of the patients with GERD were successfully treated with proton pump inhibitors (PPIs); 1 patient with URI and no history of GERD was successfully treated with antibiotics and antitussive therapy, 1 patient with left vocal cord paralysis was treated by an ENT doctor, 1 patient was treated through lifestyle improvements, and 1 patient refused all treatments because her symptoms spontaneously abated. All patients received instructions for good throat hygiene as part of their evaluation and care.

### 3.3. Follow-Up

A follow-up of all patients was conducted via telephone calls for at least 5 months after they saw a doctor. Of the 12 patients, 8 patients with GERD were treated with PPIs; 6 patients achieved complete remission, 1 patient had fewer episodes than before, and 1 patient experienced no effect. Patients without complete remission improved after the addition of ranitidine. One patient with a URI was completely relieved after treatment with antibiotics and antitussive symptomatic treatment. We suggested that this patient should undergo a GERD examination and undergo a follow-up by a GI specialist. One patient with left vocal cord paralysis was completely relieved after specialist treatment by an otorhinolaryngologist. Episodes in 1 patient were significantly reduced after lifestyle improvements, including dietary improvements, smoking and drinking cessation, and weight loss, and we suggested that this patient should also receive a GERD examination and a specialist follow-up.

## 4. Discussion

Paroxysmal laryngospasm, regardless of the etiology, is a rare event that causes considerable fear and mental stress for affected individuals. In normal swallowing, the epiglottis tilts backward and with the closure of the true and false vocal cords, food, and fluids within the pharynx are blocked from entering the airway. However, when the larynx is stimulated by either swallowed material or fluid regurgitation from the esophagus, the vocal cords are rapidly reflexively adducted, thus causing brief apnea called closed laryngeal apnea. This reaction is an appropriate, natural, and beneficial response, and, although unpleasant, resolves quickly. However, in the context of regular reflux with aspiration or other forms of irritation, a more severe form of laryngospasm with powerful and persistent closure of the larynx can occur [10]. Loughlin and Koufman reported that paroxysmal laryngospasm usually occurs in patients when they are awake [11], and Poelmans et al. indicated that paroxysmal laryngospasm occurs during the day [12]. Our study revealed that most patients experienced laryngospasm during the day, and only one experienced it at night. All patients were able to relieve their symptoms on their own, but they recurred.

In most cases, persistent recurrent paroxysmal laryngospasm is secondary to GERD as a rare complication considering the high prevalence of GERD. The process through which reflux material enters the throat is known as laryngeal regurgitation [13]. GERD is considered to be a risk factor for recurrent or persistent upper and lower respiratory diseases, including asthma, chronic cough, sinusitis, laryngitis, and serous otitis media, in addition to paroxysmal laryngospasm [14]. Knight et al. conducted a prospective study of 80 female and 32 male patients with GERD, with ages ranging from 14 to 81 years and a median age of 49.5 years. The results indicated that 4% (5/112) of the patients developed paroxysmal laryngospasm [15]. Paroxysmal laryngospasm caused by GERD may have two mechanisms: gastric juice directly injuring the mucosal surface of the upper respiratory tract, namely, the pharynx, larynx, middle ear, and sinusitis complex, which is different from the distal esophagus; the airway is not protected by the reflux clearance mechanism and inherent mucosa, and the laryngeal epithelium is very sensitive to corrosive damage of pepsin and acid. The second mechanism is activation of airway-related reflexes caused by the return of gastric contents to the distal esophagus, which is mediated by the vagus nerve [16].

Several studies have indicated that the probability of paroxysmal laryngospasm caused by GERD is approximately 80%–94%. Therefore, it is essential for patients with paroxysmal laryngospasm to undergo examinations for GERD, and active treatment should be taken immediately after diagnosis. Loughlin and Koufman conducted a prospective study of 12 consecutive adult patients with paroxysmal laryngospasm over a period of 2 years (1992–1994). Only 4 participants (33%) developed heartburn symptoms. Each patient underwent fiberoptic laryngoscopy, barium swallowing, or esophagography and 24-hour dual-probe pH monitoring (pH method). Of the 12 patients, 11 (92%) had GERD, and 10 patients (83%) had abnormal pH values. All patients responded to antireflux therapy, and the laryngospasm completely stopped after changes in diet and lifestyle [11], further adding credibility to the potential that “silent reflux” may be causing this problem in the absence of GERD symptoms. Poelmans et al. prospectively evaluated the condition of 35 patients with paroxysmal laryngospasm. Upper gastrointestinal endoscopy and pH monitoring revealed that 94% of the patients had developed gastroesophageal reflux. A few patients had laryngospasm during the examination process; the higher the frequency of laryngospasm, the higher the prevalence of esophageal hiatal hernia. Laryngospasm was completely prevented in all patients within 6 weeks after open PPI treatment and lifestyle improvement. It was also established that major otorhinolaryngological symptoms for most patients were significantly alleviated after 4 weeks of treatment with a standard dose of PPIs [12]. Obholzer et al. studied 16 patients diagnosed with paroxysmal laryngospasm and found that 80% of cases were related to GERD. PPI treatment caused complete remission of symptoms in 6 patients and partial remission of symptoms in 4 patients, and none of the patients required further treatment. Of the remaining 5 patients who did not respond to PPIs, 2 had persistent laryngospasm, and both were completely relieved after injection with laryngeal botulinum toxin [8]. The current study indicates that 8 of the 12 patients had a history of GERD, and the probability of GERD in our report was approximately 70%, which is different from the probability reported in the abovementioned previous investigation. This may be due to the small sample number of patient samples and the fact that no definite examination for gastroesophageal reflux was performed on all patients. All of our 12 patients showed chronic pharyngitis in the laryngeal physical examination, and we did not rule out gastroesophageal reflux, although no related symptoms were noted in 4 of the 12 patients. However, our data still suggested a high probability of GERD in adult paroxysmal laryngospasm.

In addition to paroxysmal laryngospasm caused by GERD, several studies have proposed other ideas. Wani and Woodson studied 6 patients with laryngeal injury and found that these patients experienced wheezing and acute airway obstruction secondary to paroxysmal laryngospasm. One of our patients would fit this etiology. Studies have also revealed that superior laryngeal nerve block or injection of botulinum toxin into the extralaryngeal thyroarytenoid muscle may be effective for temporary relief of symptoms [17]. Crumley indicated in the study that laryngospasm may be related to an abnormal recurrent laryngeal nerve and abnormal movement of the vocal cords caused by reinnervation after recurrent laryngeal nerve injury; that is, unilateral vocal cord paralysis (UVCP) can also cause paroxysmal laryngospasm [18]. Our patient with left vocal cord paralysis was completely relieved of his symptoms of paroxysmal laryngospasm after specialist treatment by an otorhinolaryngologist. Schaefer believes that viral URI is a possible etiological cause [19]. Our study indicated that several patients experienced a URI before the attack, and paroxysmal laryngospasm was relieved to some extent after the infection was cured. However, no related research is available to confirm this finding, and infection can therefore only be speculated to be the etiology. In addition, a few special cases of laryngospasm are mentioned in the literature review. Joosen et al. reported hypocalcemia-related laryngospasm leading to acute dyspnea [20]. Caietta et al. and Singh et al. reported severe recurrent laryngospasm associated with SCN4A, a rare but treatable recurrent laryngospasm caused by SCN4A mutation [21, 22].

The present treatment for paroxysmal laryngospasm in addition to the abovementioned PPI treatment for GERD, namely, omeprazole 40 mg twice a day or lansoprazole 30 mg once a day, is recommended for 3–6 months, 30 minutes before meals. A nighttime treatment with histamine-2 antagonists, such as ranitidine 300 mg or famotidine 40 mg, is also recommended for cases with poor treatment effects [7]. A clinical study by Dallemagne et al. showed that 93% and 89% of patients who underwent antireflux surgery, specifically fundoplication, showed control of their symptoms after 5 and 10 years, respectively [23]. However, this operation should not be recommended lightly and has certain surgical criteria and risks that should be evaluated via consultation with a gastroenterologist.

Wang et al. performed anastomosis of a key branch of the ansa cervicalis nerve (ACN) to the recurrent laryngeal nerve (RLN) to treat 13 patients with paroxysmal laryngospasm caused by iatrogenic therapy, including thyroid surgery, cervical surgery, and chest surgery. The airways of all patients were significantly improved after the surgical operation, and the laryngospasm disappeared completely in 92.3% (12/13) of the patients. There was no significant effect on the voice quality of patients [24]. Additionally, lifestyle changes, including improving the diet, quitting smoking and drinking, raising the head of the bed, and losing weight, may be beneficial [25].

Paroxysmal laryngospasm is a distinct phenomenon, and the current medical term for the disease is induced laryngeal obstruction (ILO). Since 2013, the term ILO has been used to describe respiratory problems caused by laryngeal spasm. This was originally proposed by the European Respiratory Association, the European Association for Laryngeal Sciences and the American Society of Thoracic Physicians [26]. The new term replaces the old term widely used to describe the disease: vocal cord dysfunction (VCD) or paradoxical vocal fold motion (PVFM). Over the years, multiple predisposing factors have been reported to be associated with PVFM [27]. Our understanding is that the new term is intended to separate the condition from the mistaken term of PVFM or laryngeal dysfunction (LD) because unlike these other conditions, ILO has a cause. The other conditions may also have some form of irritation contributing to their symptoms. However, true PVFM will not fully improve with antireflux treatment alone, and if the problem resolves with such treatment, then the condition was not PVFM. During early reporting, PVFM was mainly considered a psychological or so-called functional disorder. Currently, there are numerous reports linking GERD. A prospective study showed that 72% of PVFMD patients had laryngeal edema (90% with reflux symptoms). This regurgitation and edema may in some cases cause mild PVFMD and in others may cause complete laryngeal spasm [28]. Further studies have shown that mental aspects are more likely to be an additional factor in some PVFMD patients rather than a primary cause [29]. Although abnormal vocal cord movement in some patients may represent conversion disorders, most patients have other related causes, such as irritant exposure, GERD, exercise intolerance, and viral illness [30, 31]. Therefore, respiratory physicians should accurately differentiate the diagnosis of hysterical stridor, reflux-related laryngospasm, and asthma and thereby avoid delayed diagnosis and unnecessary ICU admission, intubation, and even surgical airway surgery. Increased familiarity with the disease characteristics, assessment, diagnosis, and management may lead to better patient outcomes and reduced healthcare spending [32]. Our data strongly support this distinction since all 12 patients who had very distressing symptoms were able to experience relief through relatively minor measures, with only one patient requiring surgery, and none of the patients required intubation or tracheostomy.

Hysterical stridor, which is known by more than 40 names in the medical literature, including “PVFM” and “LD,” is perhaps the most important condition to compare with our findings. Hysterical stridor is a functional disease characterized by inspiratory stridor. In addition, expiratory wheezing, dyspnea, shortness of breath, cough, hoarseness, voice loss, dysphagia, neck muscle tension, and throat tightness have been reported [33, 34]. These symptoms are usually similar to asthma and reflux-related laryngospasm. However, reflux-related laryngospasm leads to complete airway obstruction and interferes with inhalation and exhalation, and hysterical stridor does not respond to standard treatments for asthma. Furthermore, hysterical stridor does not appear during sleep, and nocturnal asthma attacks are common. Reflux-related laryngospasm, though often presenting in the daytime, also occurs at night. Hysterical stridor symptoms last longer than those of reflux-related laryngospasm and have been reported to last for minutes or more; the symptoms abate when the patient is alone or quiet. Hysterical stridor represents the somatic expression of an unconscious restless mood, known as a conversion disorder. By subconscious changes in their breathing patterns, they are able to manipulate their environment to provide much needed attention [35]. The disease is more common in women than in men and in adolescents and young people than in elderly individuals. These patients usually maintain normal oxygen saturation unless they have a potential lung disease. Chest X-ray and neck soft tissues are normal [36]. Pulmonary function tests during symptomatic attacks reveal chest airway obstruction or normal findings, and laryngoscopy shows paradoxical vocal cord movement, that is, vocal cord adduction during inhalation and possibly excessive vocal cord adduction during exhalation [37]. Psychological assessment usually reveals multiple sources of life stress, compulsive personality traits, depression, anxiety, maladjustment, or a history of psychosomatic comorbidity. Other psychotherapy interventions, such as antianxiety therapy, depression therapy, sedation therapy, speech therapy, and behavioral therapy, are usually effective [38, 39]. Both problems can coexist, and treating the irritation-based component with antireflux medication can improve the manageability of the other, substantially more intractable problem. A trial of this type involves no risk of exacerbating a patient's condition and will only result in improvement. Therefore, acid suppression and dietary changes, such as avoiding caffeine, carbonated beverages, citrus, chocolate, ethanol, and late-evening eating, are all worthy of consideration. However, treatment usually needs to be evaluated by physicians in the fields of pneumology, otolaryngology, gastroenterology, psychology, and language pathology [40].

Some limitations of the study should be considered. The sample size of this study was small. All patients did not undergo a GERD diagnostic examination, and we mainly relied on medical histories and exclusive identification. Our study was concluded by telephone follow-up, and the time frame was limited. Our observations may therefore be strengthened by prospective studies with larger participant groups.

## 5. Conclusion

In summary, paroxysmal laryngospasm is a rare laryngeal disease that generally occurs secondary to GERD, and antireflux or antacid therapy is effective. A few patients with paroxysmal laryngospasm largely complain of dyspnea first diagnosed in the respiratory clinic, and a respiratory physician should master and identify the symptoms and differentiate this condition from hysterical stridor, reflux-related laryngospasm, and asthma. Timely referrals to otolaryngologists, gastroenterologists, and other specialists for standardized examination and regular treatment should be made when necessary.

## Figures and Tables

**Table 1:** Patient-related information.

Number	Gender	Age	Smoking history	Drinking history	Trigger	Time of attack	History of GERD	Treatment	Outcome
1	Female	50	No	No	Cough	Daytime	No	No (treatment refusal)	Spontaneously resolved
2	Male	61	Yes	Yes	URI	Daytime	Yes	PPI	Partial relief
3	Female	45	No	No	Cough	Daytime	Yes	PPI	Complete relief
4	Male	41	No	No	URI	Daytime	Yes	PPI	Complete relief
5	Female	36	No	No	Cough	Night	Yes	PPI	Complete relief
6	Female	70	No	No	Cough	Daytime	No, but had acid regurgitation	PPI	Complete relief
7	Male	44	Yes	No	No	Daytime	Yes	PPI	Invalid
8	Female	33	No	No	Agitation	Daytime	Yes	PPI	Complete relief
9	Male	59	Yes	Yes	Agitation	Daytime	No	Improved lifestyle	Partial relief after lifestyle changes
10	Female	66	No	No	No	Daytime	No, but left vocal cord paralysis	Treatment at the Otorhinolaryngology Department	Complete relief
11	Male	31	No	No	URI	Daytime	No	Symptomatic treatment and antibiotics	Complete relief
12	Female	55	No	No	Cough	Daytime	Yes	PPI	Complete relief

GERD, gastroesophageal reflux disease; PPI, proton pump inhibitor; URI, upper respiratory tract infection.

## Data Availability

The data used to support the findings of this study are available from the corresponding author upon request.

## References

[1] Fröhlich G., Schorn K., Fröhlich H. (2020). Dyspnoe. *Der Internist*.

[2] Barnes H., McDonald J., Smallwood N., Manser R. (2016). Opioids for the palliation of refractory breathlessness in adults with advanced disease and terminal illness. *Cochrane Database of Systematic Reviews*.

[3] Weber C. K., Miglioranza M. H., Moraes M. A. (2014). The five-point Likert scale for dyspnea can properly assess the degree of pulmonary congestion and predict adverse events in heart failure outpatients. *Clinics*.

[4] Lee J. H., Lee J. H., Lee M. H., Cho H. O., Park S. E. (2017). Postoperative negative pressure pulmonary edema following repetitive laryngospasm even after reversal of neuromuscular blockade by sugammadex: a case report. *Korean Journal of Anesthesiology*.

[5] Collins S., Schedler P., Veasey B., Kristofy A., McDowel M. (2019). Prevention and treatment of laryngospasm in the pediatric patient: a literature review. *AANA Journal*.

[6] Gdynia H.-J., Kassubek J., Sperfeld A.-D. (2006). Laryngospasm in neurological diseases. *Neurocritical Care*.

[7] Holley D., Mendez A., Donald C. (2019). Paroxysmal laryngospasm: episodic closure of the upper airway. *Journal of the American Academy of Physician Assistants*.

[8] Obholzer R. J., Nouraei S. A. R., Ahmed J., Kadhim M. R., Sandhu G. S. (2008). An approach to the management of paroxysmal laryngospasm. *The Journal of Laryngology & Otology*.

[9] Maceri D. R., Zim S. (2001). Laryngospasm: an atypical manifestation of severe gastroesophageal reflux disease (GERD). *The Laryngoscope*.

[10] Rutt A. L., Bojaxhi E., Torp K. D. (2020). Management of refractory laryngospasm. *Journal of Voice*.

[11] Loughlin C. J., Koufman J. A. (1996). Paroxysmal laryngospasm secondary to gastroesophageal reflux. *The Laryngoscope*.

[12] Poelmans J., Tack J., Feenstra L. (2004). Paroxysmal laryngospasm: a typical but underrecognized supraesophageal manifestation of gastroesophageal reflux?. *Digestive Diseases and Sciences*.

[13] Ciorba A., Bianchini C., Zuolo M. (2015). Upper aerodigestive tract disorders and gastro-oesophageal reflux disease. *World Journal of Clinical Cases*.

[14] Önal Z., Çullu-Çokuğraş F., Işıldak H. (2015). Evaluation of the likelihood of reflux developing in patients with recurrent upper respiratory infections, recurrent sinusitis or recurrent otitis seen in ear-nose-throat outpatient clinics. *The Turkish Journal of Pediatrics*.

[15] Knight R. E., Wells J. R., Parrish R. S. (2000). Esophageal dysmotility as an important co-factor in extraesophageal manifestations of gastroesophageal reflux. *The Laryngoscope*.

[16] Poelmans J., Tack J. (2005). Extraoesophageal manifestations of gastro-oesophageal reflux. *Gut*.

[17] Wani M. K., Woodson G. E. (1999). Paroxysmal laryngospasm after laryngeal nerve injury. *The Laryngoscope*.

[18] Crumley R. L. (2000). Laryngeal synkinesis revisited. *Annals of Otology, Rhinology & Laryngology*.

[19] Schaefer S. D. (1983). Neuropathology of spasmodic dysphonia. *The Laryngoscope*.

[20] Joosen D. A. W. A., Laar R. J. J. M. V. D., Koopmans R. P., Stassen P. M. (2015). Acute dyspnea caused by hypocalcemia-related laryngospasm. *The Journal of Emergency Medicine*.

[21] Caietta E., Milh M., Sternberg D. (2013). Diagnosis and outcome of SCN4A-related severe neonatal episodic laryngospasm (SNEL): 2 new cases. *Pediatrics*.

[22] Singh R. R., Tan S. V., Hanna M. G., Robb S. A., Clarke A., Jungbluth H. (2014). Mutations in SCN4A: a rare but treatable cause of recurrent life-threatening laryngospasm. *Pediatrics*.

[23] Dallemagne B., Weerts J., Markiewicz S. (2006). Clinical results of laparoscopic fundoplication at ten years after surgery. *Surgical Endoscopy*.

[24] Wang W., Sun J., Tang H. (2019). Main branch of ACN-to-RLN for management of laryngospasm due to unilateral vocal cord paralysis. *The Laryngoscope*.

[25] Gupta R., Sataloff R. T. (2009). Laryngopharyngeal reflux: current concepts and questions. *Current Opinion in Otolaryngology & Head and Neck Surgery*.

[26] Christensen P. M., Heimdal J.-H., Christopher K. L. (2015). ERS/ELS/ACCP 2013 international consensus conference nomenclature on inducible laryngeal obstructions. *European Respiratory Review*.

[27] Sayad E., Das S. (2020). *Exercise Induced Laryngeal Obstruction (Vocal Cord Dysfunction)*.

[28] Forrest L. A., Husein T., Husein O. (2012). Paradoxical vocal cord motion: classification and treatment. *The Laryngoscope*.

[29] Boulet L.-P. (2012). Cough and upper airway disorders in elite athletes: a critical review. *British Journal of Sports Medicine*.

[30] Morris M. J., Christopher K. L. (2010). Diagnostic criteria for the classiria for the classisi classiitical. *Chest*.

[31] Franca M. C. (2014). Differential diagnosis in paradoxical vocal fold movement (PVFM): an interdisciplinary task. *International Journal of Pediatric Otorhinolaryngology*.

[32] Denipah N., Dominguez C. M., Kraai E. P., Kraai T. L., Leos P., Braude D. (2017). Acute management of paradoxical vocal fold motion (vocal cord dysfunction). *Annals of Emergency Medicine*.

[33] Lacy T. J., McManis S. E. (1994). Psychogenic stridor. *General Hospital Psychiatry*.

[34] Butani L., O’Connell E. J. (1997). Functional respiratory disorders. *Annals of Allergy, Asthma & Immunology*.

[35] Snyder H. S., Weiss E. (1989). Hysterical stridor: a benign cause of upper airway obstruction. *Annals of Emergency Medicine*.

[36] Matrka L. (2014). Paradoxic vocal fold movement disorder. *Otolaryngologic Clinics of North America*.

[37] Tousignant G., Kleiman S. J. (1992). Functional stridor diagnosed by the anaesthetist. *Canadian Journal of Anaesthesia*.

[38] Liao K. S., Kwak P. E., Hewitt H., Hollas S., Ongkasuwan J. (2017). Measuring quality of life in pediatric paradoxical vocal fold motion using the SF-36v2. *Journal of Voice*.

[39] Loveleen G., Atkinson S., Avinash H., Guglani L. (2014). A systematic review of psychological interventions for adult and pediatric patients with vocal cord dysfunction. *Frontiers in Pediatrics*.

[40] Ali A.-A., David K. (2012). Vocal cord dysfunction in athletes: clinical presentation and review of the literature. *The Physician and Sportsmedicine*.

